# Identification of prognostic stemness-related genes in kidney renal papillary cell carcinoma

**DOI:** 10.1186/s12920-024-01870-2

**Published:** 2024-05-03

**Authors:** Yifan Liu, Yuntao Yao, Yu Zhang, Chengdang Xu, Tianyue Yang, Mingyu Qu, Bingnan Lu, Xu Song, Xiuwu Pan, Wang Zhou, Xingang Cui

**Affiliations:** 1 Department of Urology, Xinhua Hospital Affiliated to Shanghai Jiao Tong University School of Medicine, No.1665 Kongjiang Road, Shanghai, 200092 China; 2 Shanghai Jiao Tong University School of Medicine, Shanghai, 200025 China; 3 Tongji University School of Medicine, Shanghai, 200092 China; 4 Department of Urology, Shanghai Seventh People’s Hospital, Shanghai, Shandong 200137 China

**Keywords:** Kidney renal papillary cell carcinoma (KIRP), Stemness-related gene, mRNA expression-based stemness index, Notch signaling pathway, Prognosis, Target drugs

## Abstract

**Background:**

Kidney renal papillary cell carcinoma (KIRP) is the second most prevalent malignant cancer originating from the renal epithelium. Nowadays, cancer stem cells and stemness-related genes (SRGs) are revealed to play important roles in the carcinogenesis and metastasis of various tumors. Consequently, we aim to investigate the underlying mechanisms of SRGs in KIRP.

**Methods:**

RNA-seq profiles of 141 KIRP samples were downloaded from the TCGA database, based on which we calculated the mRNA expression-based stemness index (mRNAsi). Next, we selected the differentially expressed genes (DEGs) between low- and high-mRNAsi groups. Then, we utilized weighted gene correlation network analysis (WGCNA) and univariate Cox analysis to identify prognostic SRGs. Afterwards, SRGs were included in the multivariate Cox regression analysis to establish a prognostic model. In addition, a regulatory network was constructed by Pearson correlation analysis, incorporating key genes, upstream transcription factors (TFs), and downstream signaling pathways. Finally, we used Connectivity map analysis to identify the potential inhibitors.

**Results:**

In total, 1124 genes were characterized as DEGs between low- and high-RNAsi groups. Based on six prognostic SRGs (CCKBR, GPR50, GDNF, SPOCK3, KC877982.1, and MYO15A), a prediction model was established with an area under curve of 0.861. Furthermore, among the TFs, genes, and signaling pathways that had significant correlations, the CBX2-ASPH-Notch signaling pathway was the most significantly correlated. Finally, resveratrol might be a potential inhibitor for KIRP.

**Conclusions:**

We suggested that CBX2 could regulate ASPH through activation of the Notch signaling pathway, which might be correlated with the carcinogenesis, development, and unfavorable prognosis of KIRP.

**Supplementary Information:**

The online version contains supplementary material available at 10.1186/s12920-024-01870-2.

## Introduction

Kidney renal papillary cell carcinoma (KIRP) is a malignancy known as the second most common histologic variant of renal cell carcinoma (RCC) and contributes to 10–15% of RCC morbidity [1, 2]. Therapeutically, targeted drugs are widely used in the treatment of advanced RCC, such as sorafenib and sunitinib [3, 4], but these drugs that target kidney renal clear cell carcinoma (KIRC) are still limited in advanced KIRP [2]. Due to the low prevalence of KIRP in RCC and the different mechanisms between KIRP and KIRC, patients with KIRP are always excluded from those large clinical trials of the current targeted therapies [5]. Therefore, it’s urgent to explore the mechanisms of KIRP and find its therapeutic targets.

Nowadays, cancer stem cells (CSCs) have been given greater attention. As we all know, CSCs are able to self-renew and differentiate, and they play important roles in tumorigenesis and metastasis [5]. The stemness index is utilized to quantify the characteristics of CSCs in tumors. The mRNA expression-based stemness index (mRNAsi) and the DNA methylation-based stemness index (mDNAsi) represent the expression of transcriptomic stemness and the features of epigenetic stemness, respectively. It has been revealed that mRNAsi is linked with the tumorigenesis and prognosis of hepatocellular carcinoma (HCC) [6]. Nevertheless, the association between mRNAsi and KIRP is still unknown.

In the present study, we collected RNA-seq profiles and clinical information of KIRP patients from The Cancer Genome Atlas (TCGA) database and characterized differentially expressed genes (DEGs) between low- and high-stemness groups. Next, we conducted a comprehensive analysis to estimate the association between stemness-related genes (SRGs) and overall survival (OS), and subsequently constructed a prognostic model. Then, we utilized weighted gene correlation network analysis (WGCNA), and univariate Cox analysis to identify prognostic SRGs. Afterwards, they were included in the multivariate Cox regression analysis, and established a prognostic model. In addition, a regulatory network was constructed by Pearson correlation analysis, which included key genes, upstream transcription factors (TFs), and downstream signaling pathways to clarify the potential mechanisms of tumorigenesis and metastasis in KIRP. Finally, we used Connectivity map (Cmap) analysis to identify the potential inhibitors targeting KIRP. Hopefully, the prognostic model might be beneficial to clinical decision-making, and the potential therapeutic targets would be useful for the treatment of KIRP patients.

## Materials and methods

### Data collection

The Ethics Committee of Xinhua Hospital Affiliated to Shanghai Jiao Tong University School of Medicine approved this study (XHEC-C-2021-145-1). We downloaded the RNA sequencing profiles and clinical information of 141 KIRP patients from TCGA database (https://tcga-data.nci.nih.gov), including demographic factors, patients’ survival, and clinical stages. The results were demonstrated in a table.

### mRNAsi estimation

One-class logistic regression machine learning (OCLR) was employed to evaluate the stemness signatures of the KIRP samples as mRNAsi, which was an index between 0 and 1 [7, 8]. Higher mRNAsi presented higher activity of CSCs in the bulk tumor tissue and greater dedifferentiation of malignant cells. In the current study, mRNAsi of samples were evaluated by OCLR utilizing a normalized gene expression matrix acquired from RNA sequencing profiles.

### Differential expression analysis for low- and high-mRNAsi groups

In this study, we deployed the limma package to find differential expression genes. All KIRP samples were categorized into two groups, low- and high-stemness groups. The high-stemness group contained samples with mRNAsi values greater than the median value, and the low-stemness group included samples with mRNAsi values less than the median value. We conducted differential expression analysis between low- and high-stemness groups with a cut-off value of *P* < 0.05 and log_2_ Fold Change (FC) > 2.0 or < -2.0 to explore the DEGs.

### Functional enrichment analysis

In order to further investigate the functions of the DEGs, the clusterProfiler package was utilized to conduct Gene Ontology (GO) and Kyoto Encyclopedia of Genes and Genomes (KEGG) functional enrichment analyses [9]. Also, we conducted Gene Set Variation Analysis (GSVA) to quantify the 50 hallmarks of cancer gene sets in each sample [10].

### Weighted gene correlation network analysis

We used the weighted gene correlation network analysis (WGCNA) package to perform the subsequent analyses [11]. The identified DEGs between low- and high-stemness groups were input for co-expression network analysis. Then, mRNAsi and quantitative signaling pathways were used as clinical phenotypes. With the gene modules, module dendrograms were constructed. In the principal component analysis of each module, the module eigengenes (MEs) possessing an expression profile signature, which was characterized by the expression patterns of all genes, constituted the main components. The module membership (MM) was determined by the association between genes in the corresponding modules and mRNAsi. After selecting modules of significance, we counted gene significance (GS) and MM for each key gene. We then chose the modules with the highest correlation factor between mRNAsi and standardized gene expression of modules for further analysis. Genes whose correlation with MM (cor.gene MM) and GS (cor.gene GS) > 0.5 were regarded as SRG.

### Construction of the prognostic model

We subsequently performed the univariate Cox regression analysis for the SRGs in the pink module (cor.gene MM > 0.5 and cor.gene GS > 0.5). And then the SRGs with prognostic significance were put into the multivariate Cox regression model. Based on the multivariate Cox regression model, we evaluated the risk score based on the following formula for each KIRP patient. The formula was:

$$\begin{aligned}\text{Risk\,score} &=\beta{1} \times \text{gene}1 + \beta{2} \times \text{gene}2 + \beta{3} \times \\&\text{gene}3\ldots\ldots + \beta{n} \times \text{genen}\end{aligned}$$

In this formula, “x” referred to the number of patients, “β” referred to the coefficient of SRGs in the multivariate model, and “n” referred to the number of SRGs in the multivariate model.

The receiver operator characteristic (ROC) curve was used to test the efficacy of the multivariate Cox regression model. After that, we divided all KIRP patients into two risk groups according to the median value of risk score. Additionally, we also constructed the Kaplan-Meier (K-M) survival analysis to estimate the prognostic value of the risk score in KIRP. We then applied the univariate and multivariate Cox regression analyses to assess the prognostic value of risk score, together with age and TNM stage.

### Construction of regulatory network

Firstly, 318 cancer-related TFs and 50 hallmarks of cancer gene sets were extracted from the Cistrome database (http://cistrome.org/) and the Molecular Signatures Database (MSigDB) v7.0 (https://www.gsea-msigdb.org/gsea/msigdb/index.jsp). Next, we selected differentially expressed TFs between primary tumors and metastatic tumors, and the significant TFs were depicted in a heatmap. Moreover, we performed co-expression analysis with differentially expressed TFs, SRGs, and 50 hallmarks of cancer. The selected criteria of the Pearson correlation analysis were|correlation coefficient| > 0.40 and *P* < 0.05. Eventually, the results were visualized as a regulatory network, which directly demonstrated the potential regulatory relationship among TF, SRGs, and the 50 hallmarks of cancer.

### Identification of candidate target drugs

By adopting the Connectivity Map (CMap) database, we aimed to identify candidate small molecules that might be potential drugs against CSCs. CMap analysis was performed with the results of differential expression analysis between high- and low-mRNAsi groups in pan-cancer reported by Tathiane M et al. as input data [7, 12, 13]. Afterwards, small molecules that were significant in more than 10 types of cancer were demonstrated in a heatmap. Additionally, chemical structures of small molecules were downloaded from the Clue database (https://clue.io/).

### Assay for transposase-accessible chromatin sequencing (ATAC-seq) and chromatin immunoprecipitation sequencing (ChIP-seq) analyses

Assay for transposase-accessible chromatin sequencing (ATAC-seq) data of KIRP samples were downloaded from the TGCA cohort of chromatin accessibility landscape of primary human cancers (https://gdc.cancer.gov/about-data/publications/ATACseq-AWG) [14]. ATAC-seq could validate the chromatin accessibility at the location of the key genes. Next, the analysis was done using the UCSC genome browser home (www.genome.ucsc.edu), and the “org.Hs.eg.db” R package and “TxDb.Hsapiens.UCSC.hg38.knownGene” R package. We divided each chromosome into 92 segments and normalized the number of binding peaks by the peak densities. Then, we counted the total number of peaks in those segments, to characterize the chromatin accessibility for the corresponding region, helping validate the chromatin accessibility of the key regulatory genes.

Moreover, to validate the potential regulatory relationship between the key TF and target gene, ChIP-seq profiles of two samples (GSM2700104 and GSM2700105) [15] in “bigwig” format were downloaded from the Cistrome Data Browser (DB) (http://cistrome.org/db/#/) [16]. Afterwards, the integrative genomics viewer (IGV) was used to visualize the result [17].

### Multidimensional validation

In order to reduce bias, we used multiple databases, including K-M plotter [18], Gene Expression Profiling Interactive Analysis (GEPIA) [19], LinkedOmics [20], UALCAN [21], the Human Protein Atlas [22], and single-cell RNA sequencing analysis [23, 24] to evaluate gene and protein expression levels of critical biomarkers at the tissue and cellular levels.

### Single-cell RNA-sequencing validation

A total of 31,390 single-cell RNA sequencing (scRNA-seq) profiles were downloaded from the National Center for Biotechnology Information Gene Expression Omnibus (NCBI-GEO) (accession no.GSE159115) [25, 26], which were composed of 11,740 from six benign adjacent kidney tissue (BA) samples and 19,650 from eight renal cell carcinoma (RCC) samples. Next, we conducted t-distributed stochastic neighbor embedding (tSNE) analysis for dimensional reduction. Then, we used the marker genes to annotate each cell spot, dividing the cells into six subclasses (B cells, endothelial cells, epithelial cells, fibroblasts, myeloid cells, and NK/T cells). Afterwards, we demonstrated the cell proportion of each sample, and the top five marker genes of each subtype. Finally, we demonstrated the significant gene (ASPH), TF (CBX2), and five important genes in the Notch signaling pathway.

### Statistics analysis

In this study, results with *P* < 0.05 were thought to be statistically significant. All statistical analyses were conducted by R version 3.6.1 software (Institute for Statistics and Mathematics, Vienna, Austria; www.r-project.org) deploying the limma, Seurat, ggplot2, SingleR, reticulate, clusterProfiler, GSEABase, and GSVA packages.

## Result

### Differential gene expression analysis

**Fig. 1:**
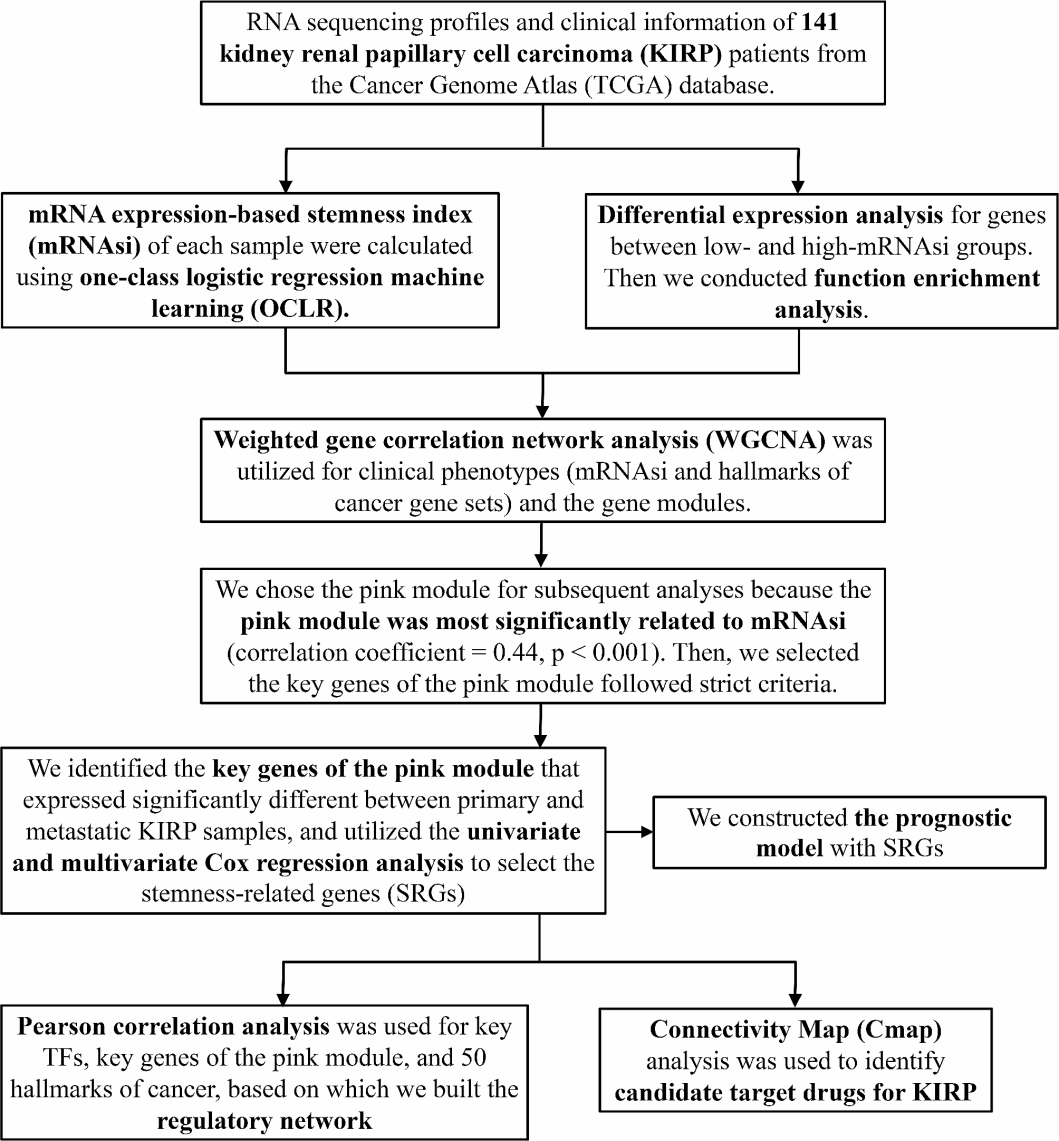
The flowchart of our analysis processes.

**Fig. 2:**
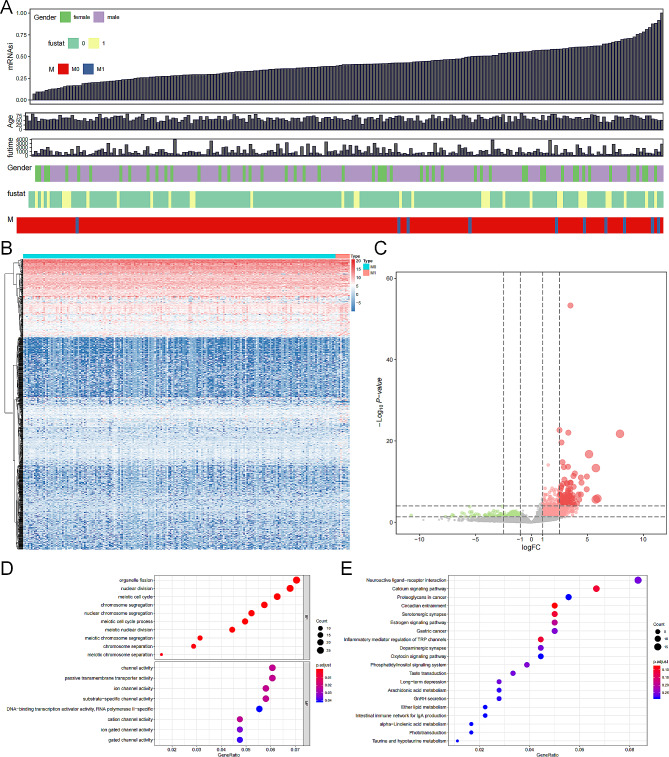
The mRNAsi of the KIRP samples, the results of differential gene expression analysis between high- and low-mRNAsi samples in KIRP, and functional enrichment analysis. (**A**) The mRNAsi summary of all samples, which was calculated through OCLR. (**B**) The heatmap of the differential expressed genes between high- and low-mRNAsi samples. (**C**) The volcano plot of the DEGs. (**D**) The bubble plot of GO analysis in BP and MF. (**E**) The bubble plot of KEGG pathways enrichment analysis. *Abbreviations:* mRNAsi, mRNA expression-based stemness index; KIRP, kidney renal papillary carcinoma; OCLR, one-class logistic regression machine learning; DEG, differentially expressed genes; BP, biological process; MF, molecular function.

We presented the flowchart of each analysis process in this study in Fig. 1 and the baseline clinical characteristics of KIRP patients in Table 1; Fig. 2A. Totally, there were 1,124 genes identified as DEGs between high- and low-mRNAsi groups, with 959 upregulated and 165 downregulated genes included. The heatmap and volcano plot were respectively presented in Fig. 2B and C.

**Table 1:** Baseline characteristics of 141 patients diagnosed with KIRP

Variables	Total patients (*N* = 141)
**Age, years**	
Mean ± SD	59.84 ± 12.33
Median (Range)	60.00(28.00–85.00)
**Survival month**	
Mean ± SD	975.33 ± 797.26
Median (Range)	740.00 (3.00–3950.00)
**Clinical Stage**	
Stage I	92 (65.25%)
Stage II	16 (11.35%)
Stage III	23 (16.31%)
Stage IV	10 (7.09%)
**Clinical T**	
T1	98 (69.50%)
T2	20 (14.18%)
T3	25 (17.73%)
T4	1 (0.71%)
**Clinical N**	
N1	122 (86.52%)
N2	18 (12.77%)
N3	1(0.71%)
**Clinical M**	
M1	132 (93.62%)
M2	9 (6.38%)

*Abbreviations* SD, standard deviation; KIRP, kidney renal papillary carcinoma

### Functional enrichment analysis

Functional enrichment analyses on the DEGs in GO terms and KEGG pathways were exhibited in Fig. 2D and Fig. 2E. The GO enrichment analysis revealed that the DEGs were enriched in biological processes including “organelle fission”, “nuclear division”, “meiotic cell cycle” and “chromosome segregation”, as well as terms of molecular function such as “channel activity”, “passive transmembrane transporter activity”, and “ion channel activity”. The KEGG enrichment analysis also showed significantly enriched pathways, including “neuroactive ligand-receptor interaction”, “calcium signaling pathway”, and “proteoglycans in cancer”.

### Weighted gene correlation network analysis

**Fig. 3:**
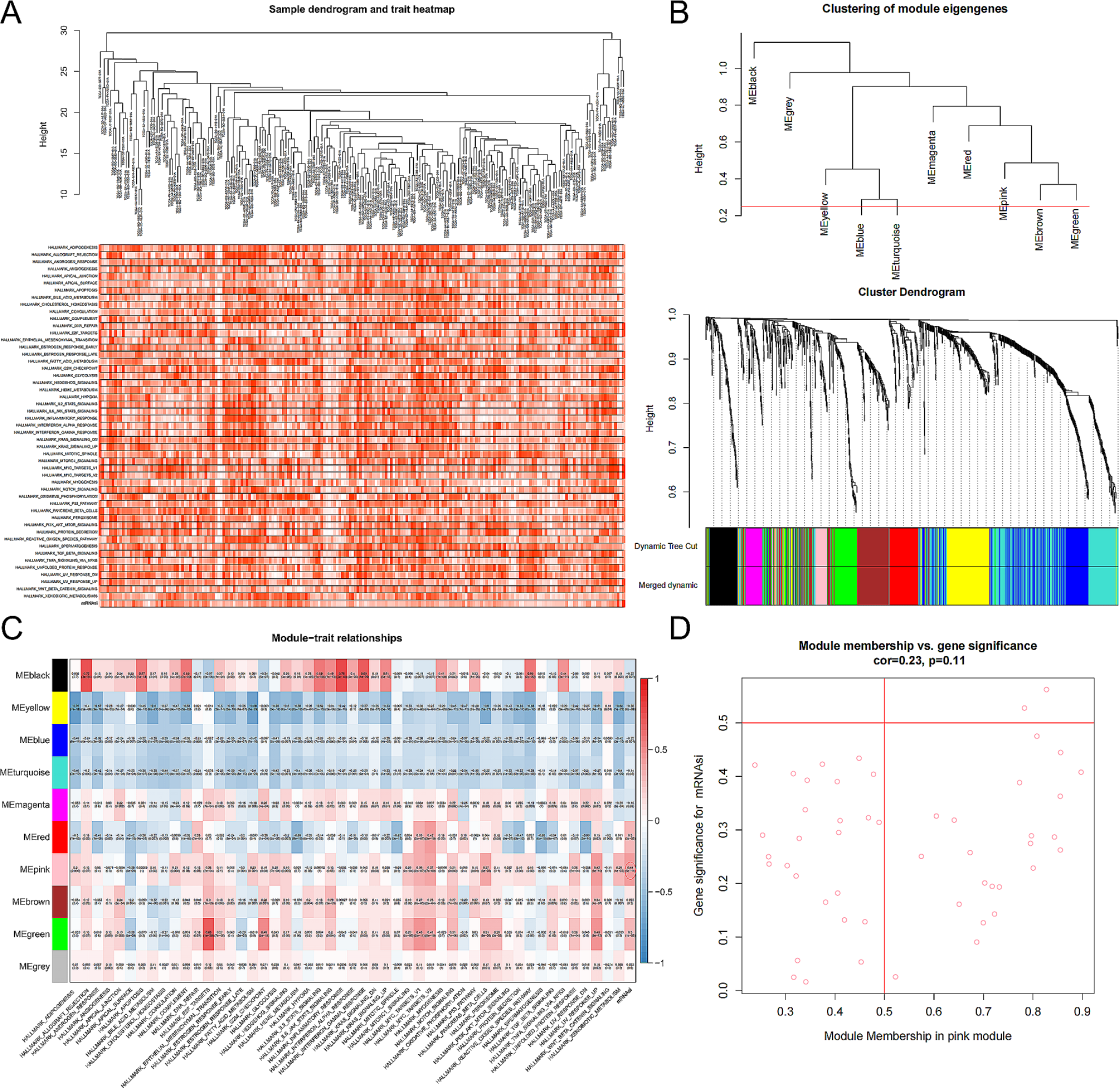
Weighted gene co-expression network analysis for the clinical phenotypes and the DEGs. (**A**) Samples dendrogram and trait heatmap. (**B**) Clustering of module eigengenes and cluster dendrogram identified different modules (black, yellow, blue, turquoise, magenta, red, pink, brown, green, and gray). (**C**) Pearson correlation analysis between the gene module and 50 hallmarks of cancer and mRNAsi. The correlation coefficient and corresponding P value are annotated. (**D**) Scatter plot of module eigengenes in the pink module for mRNAsi. *Abbreviations:* DEG, differentially expressed genes; mRNAsi, mRNA expression-based stemness index.

Sample dendrogram and trait heatmap by WGCNA were illustrated in Fig. 3A. And the identification of co-expression modules in KIRP was demonstrated in Fig. 3B. In order to evaluate the relationships between the mRNAsi of the samples and the modules, the expression level of the overall gene that belonged to the corresponding module was calculated by MS. Then, MS was used to evaluate the correlations with clinical phenotypes. The pink module was most significantly related to mRNAsi (correlation coefficient = 0.44, *P* = 6e^− 11^, Fig. 3C). Therefore, we selected the pink module as the module of interest for subsequent analyses. We defined the threshold for selecting the key genes in the mRNAsi group as cor. GS > 0.5 and cor. MM > 0.5 (Fig. 3D).

### Calculation of risk score and independent prognostic analysis

**Fig. 4:**
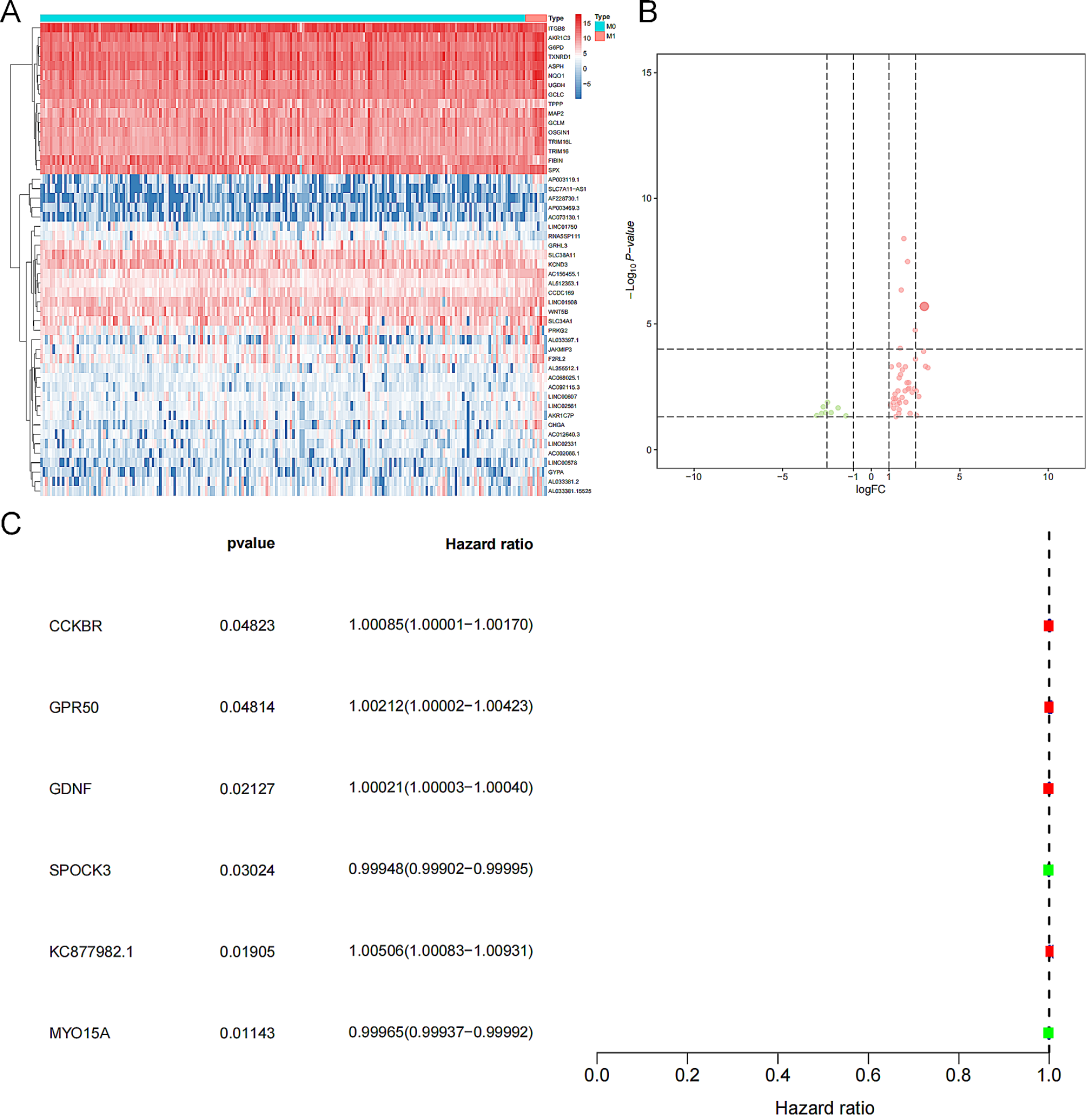
The SRGs of the pink module and the univariate Cox regression analysis. (**A**) The heatmap of the SRGs in the primary and metastatic samples. (**B**) The volcano plot of the SRGs. (**C**) Univariate Cox regression analysis identified the prognostic SRGs, which was visualize by the forest map. *Abbreviations:* SRGs, stemness-related genes.

**Fig. 5:**
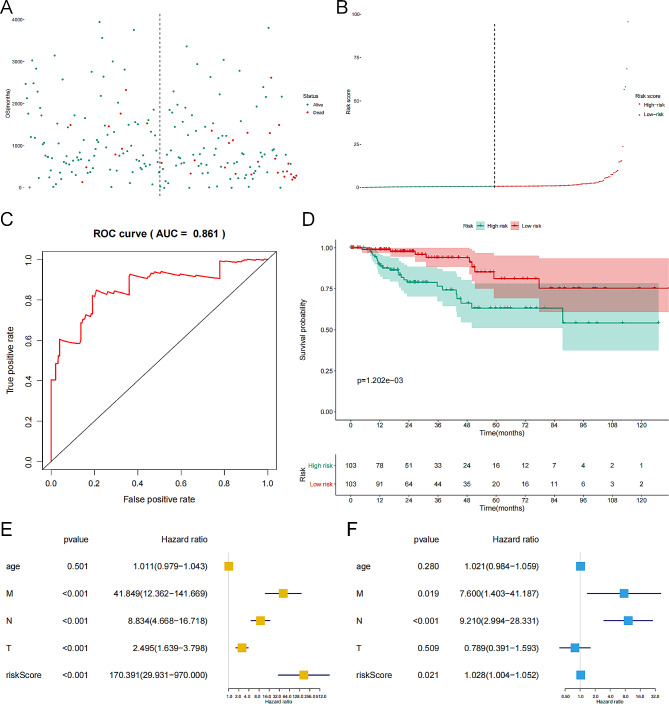
Model diagnosis of our prognostic model, and multivariate Cox regression analysis helped identify the independent prognostic value of the risk score. (**A**) The risk scatter of all samples and the overall survival profiles of all KIRP patients. (**B**) The risk score curve of all KIRP patients. (**C**) The ROC curve for evaluating the reliability of the predictive model. (**D**) The K-M survival analysis validated significantly different survival probability for the patients with low risk score and high risk score. (**E**) The univariate Cox regression analysis of risk score, age and TNM stage. (**F**) The multivariate Cox regression analysis of risk score, age and TNM stage. *Abbreviations:* KIRP, kidney renal papillary carcinoma; ROC, receiver operator characteristic curve.

We displayed the expression level of key genes (cor. GS > 0.5 and cor. MM > 0.5) of the pink module in the primary tumors and the metastatic tumors with a heatmap and a volcano plot (Fig. 4A, 4B), which were named as SRGs. Six SRGs had prognostic significance in the univariate Cox regression analysis (Fig. 4C). We incorporated these SRGs into a multivariate Cox model and then calculated the riskscore of each KIRP patient. The distribution of all KIRP patients’ riskscore was illustrated in the scatter plot and the risk curve (Fig. 5A, 5B). In addition, the receiver operator characteristic curve (ROC) curve illustrated the modest fitness and accuracy of the multivariate Cox regression model (area under curve (AUC) = 0.861) and K-M survival curve revealed the significant prognostic value of the riskscore (*P* < 0.001) (Fig. 5C, 5^5^D). We subsequently confirmed that the risk score was an independent prognostic predictor for KIRP in univariate (hazard ratio (HR) = 170.391, 95% confidential interval (CI) (29.931–970.000), *P* < 0.001, Fig. 5E) and multivariate Cox regression analysis (HR = 1.028, 95%CI (1.004–1.052), *P* = 0.021), Fig. 5F).

### Construction of the regulatory network

**Fig. 6:**
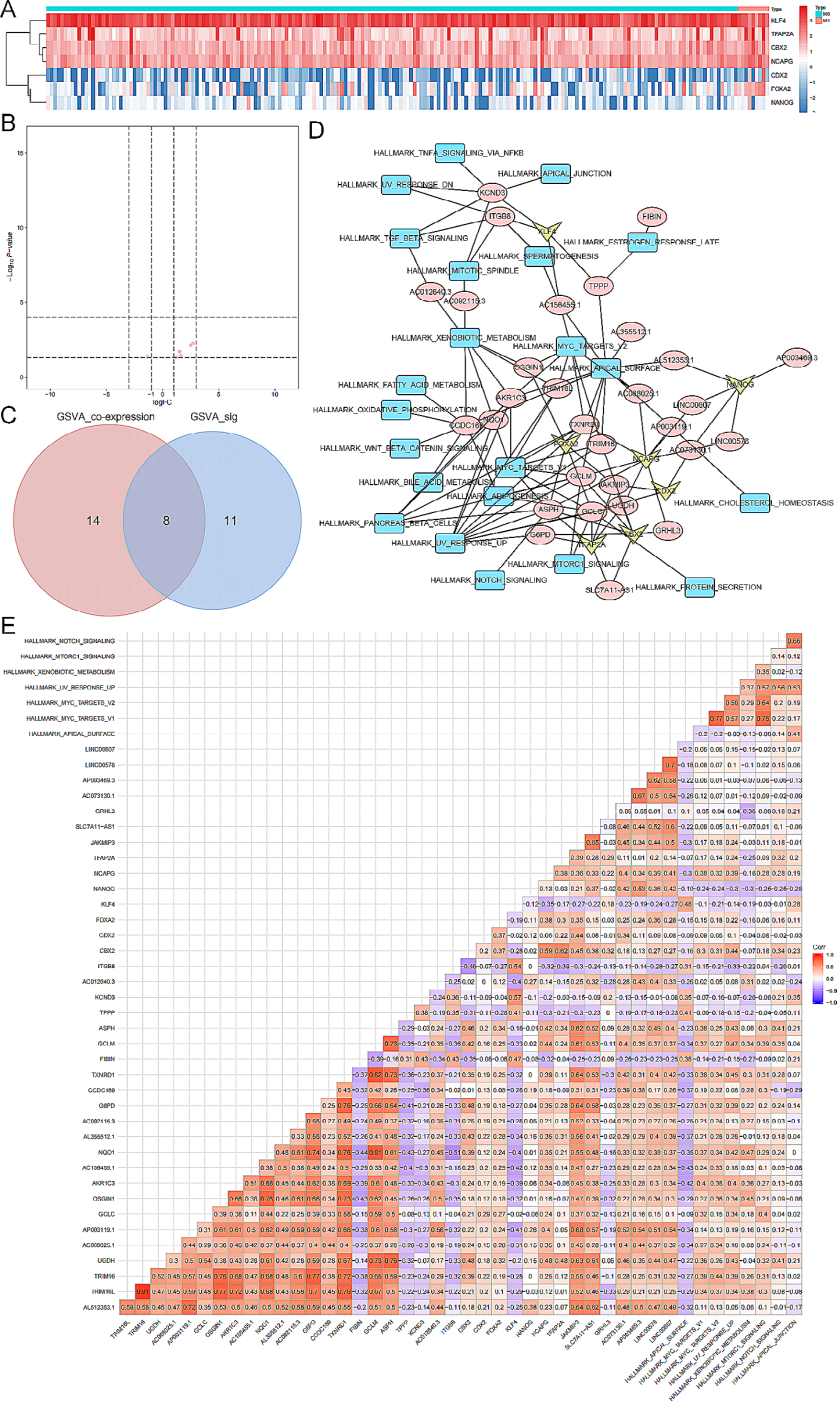
Pearson correlation analysis identified the significant correlation among significant TFs, SRGs and signaling pathways, on the basis of which we built a regulatory network. (**A**) The heatmap of the significant TFs (CBX2, CDX2, FOXA2, KLF4, NANOG, NCAPG, and TFAP2A). (**B**) The volcano plot of the significant TFs. (**C**) The Venn plot demonstrated that 8 signaling pathways were significant in both sets. (**D**) The regulatory network of significant TFs, SRGs and signaling pathways. (**E**) The heatmap of the correlation factor among significant TFs, SRGs and signaling pathways. *Abbreviations:* TF, transcription factor; SRG, stemness-related genes.

Firstly, we performed the TF enrichment analysis between primary tumors and metastatic tumors, and finally, seven TFs were identified as differentially expressed TFs, including CBX2, CDX2, FOXA2, KLF4, NANOG, NCAPG, and TFAP2A, which was demonstrated in a heatmap (Fig. 6A) and a volcano plot (Fig. 6B). What’s more, differential expression analysis identified 19 significant signaling pathways between primary and metastatic KIRP samples, and 22 signaling pathways were significantly co-expressed with the pink module in the WGCNA. Finally, intersecting the two sets (Fig. 6C), eight signaling pathways were selected, including Notch signaling pathway, Apical surface pathway, MYC targets V1 pathway, MYC targets V2 pathway, UV response up pathway, Xenobiotic metabolism pathway, mTORC1 signaling pathway, and Apical junction pathway.

Furthermore, we performed Pearson correlation analysis among significantly enriched seven TFs, SRGs of the pink module, and 50 hallmarks of cancer. As a result, 115 co-expression interaction pairs were used to construct the regulation network (Fig. 6D). What’s more, a co-expression heatmap was also exhibited to present the co-expression patterns of 115 interaction pairs (Fig. 6E). Based on the co-expression network, we found that the CBX2-ASPH-Notch signaling pathway axis was the most significant, implying its potential role in promoting the metastasis of KIRP.

### Identification of candidate target drugs

**Fig. 7:**
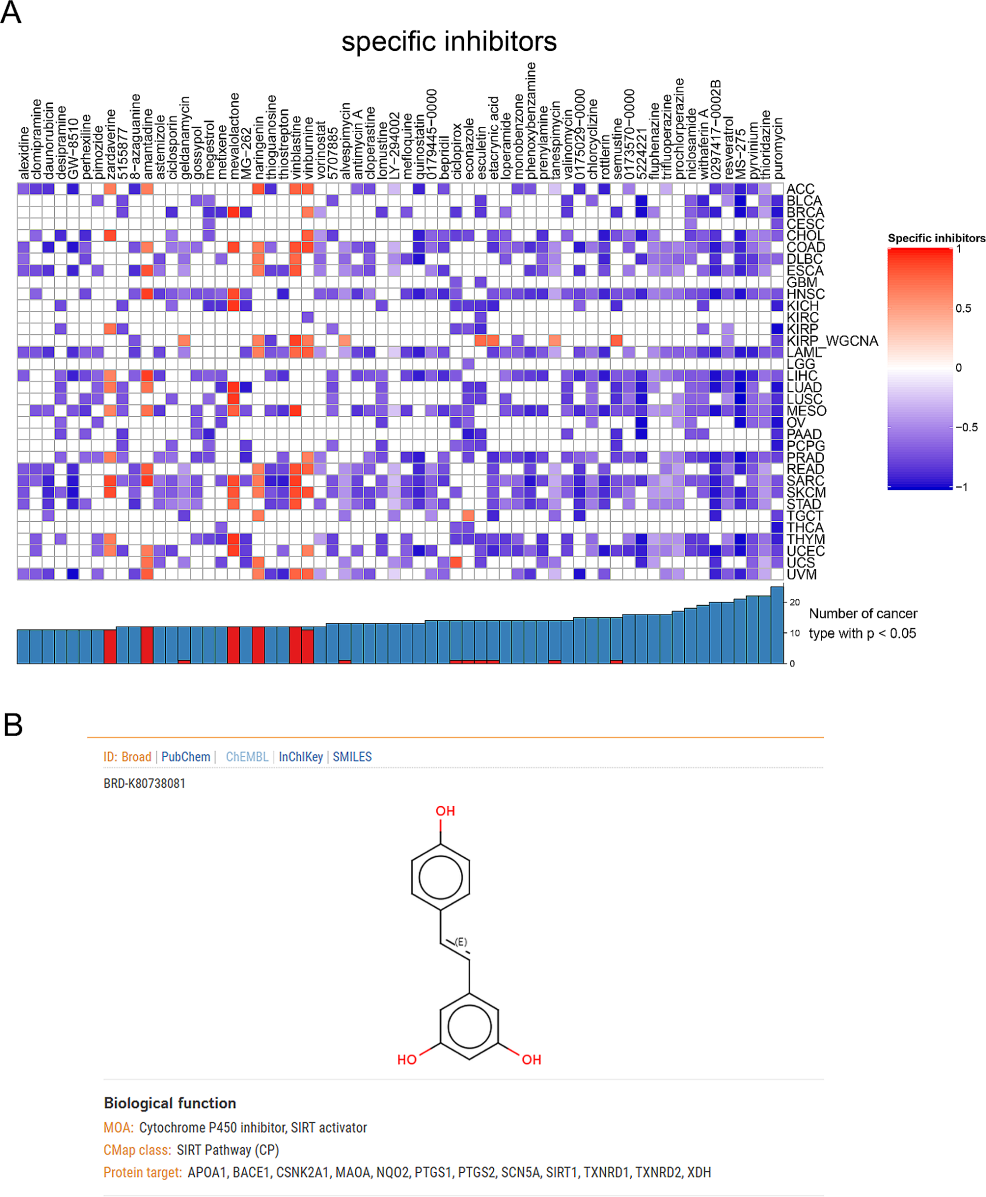
The Cmap analysis of special inhibitors targeting the KIRP. (**A**) The heatmap of potential candidate drugs across cancers. (**B**) The chemical structures details of resveratrol. *Abbreviations:* Cmap, Connectivity map; KIRP, kidney renal papillary cell carcinoma.

We used the DEG analysis results between high- and low-mRNAsi samples in KIRP as input data to do CMap analysis. A heatmap was constructed based on small molecules whose statistical results were significant in more than 10 kinds of cancers (Fig. 7A). The result proved that puromycin, resveratrol, semustine, tanespimycin, monobenzone, etacrynic acid, esculetin, alvespimycin, vinburnine, vinblastine, thioguanosine, naringenin, geldanamycin, and 8-azaguanine were potential small molecules that targeted SRGs and TFs in KIRP. After consulting current literatures, we found that the most significant potential molecule was resveratrol. Additionally, the chemical structure of resveratrol was also downloaded from the clue database (https://clue.io/) (Fig. 7B).

### ATAC-seq and ChIP-seq analyses

**Fig. 8:**
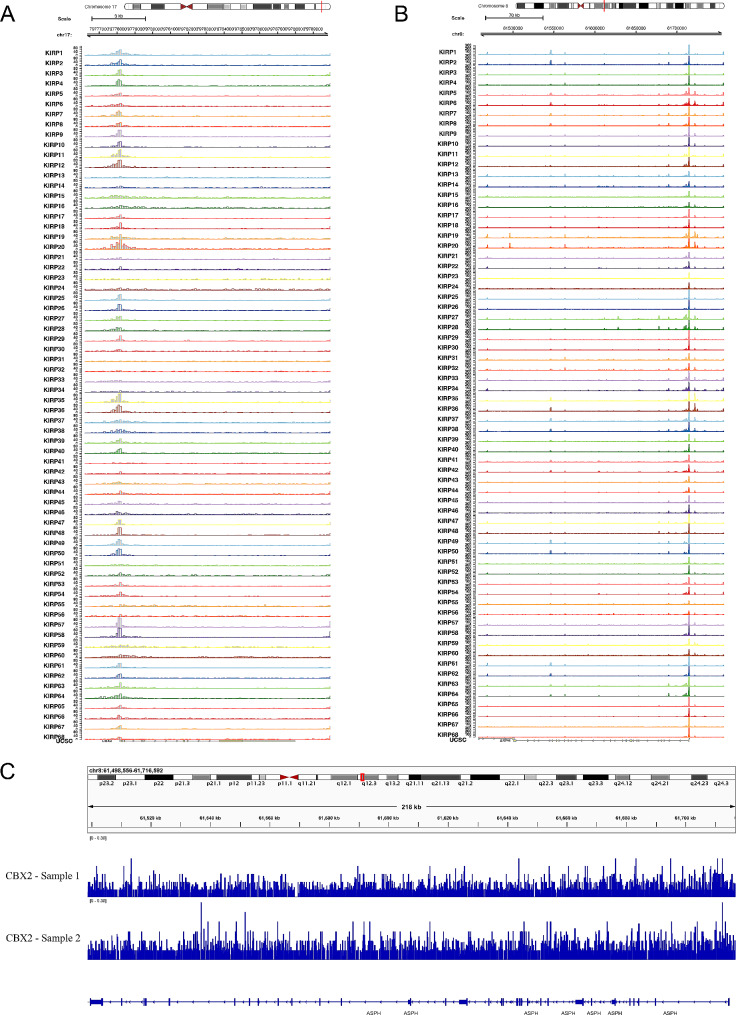
ATAC-seq and ChIP-seq validation (**A**) ATAC-seq helped validate the chromatin accessibility at the location of CXB2. (**B**) ATAC-seq helped validate the chromatin accessibility at the location of ASPH. (**C**) Bnding peaks of CXB2 were found in ASPH sequence (GRCh38/hg38, chromosome 8: 61,498,556 − 61,716,592) in two ChIP-seq samples, and the binding motifs of CXB2 were predicted *Abbreviations:* ATAC-seq, Assay for Transposase-Accessible Chromatin Sequencing; ChIP-seq, Chromatin Immunoprecipitation Sequencing.

In Fig. 8A-B, ATAC-seq helped validate the chromatin accessibility at the location of the key genes (CXB2, ASPH). The peaks of the chromosomes suggested active transcription processes happened, so that we could further evidence the critical roles of CXB2 and ASPH in regulating KIRP. What’s more, in ChIP-seq validation, binding peaks of CXB2 were found in ASPH sequence (GRCh38/hg38, chromosome 8: 61,498,556 − 61,716,592) in two ChIP-seq samples (Fig. 8C), and the binding motifs of CXB2 were predicted. Taken together, we further proved a potential transcriptional regulation pattern between CXB2 and ASPH to regulate Notch signaling pathway in KIRP.

### Multidimensional validation

**Fig. 9:**
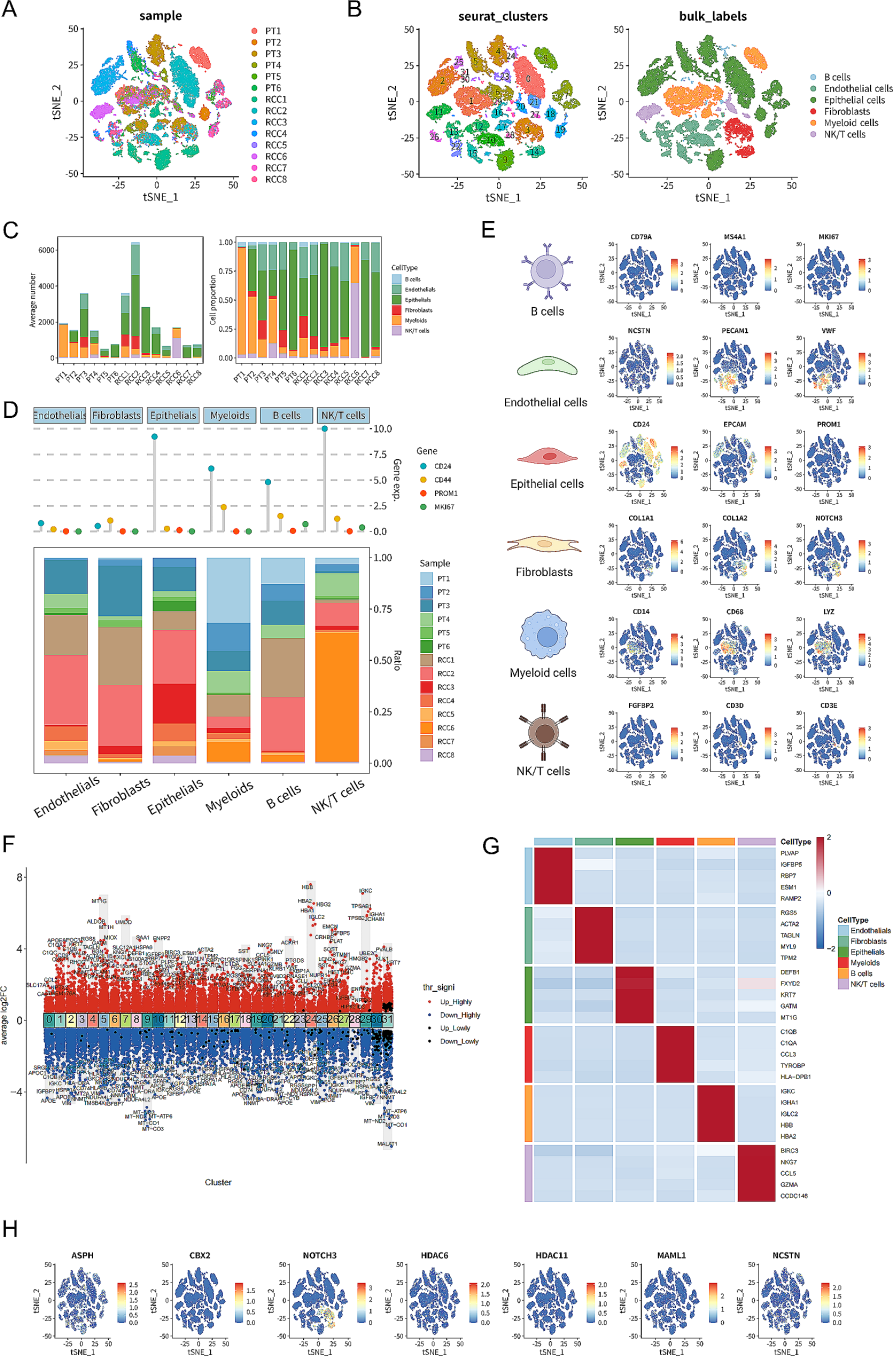
scRNA-seq validation. (**A**) tSNE dimension reduction analysis for 14 samples. (**B**) tSNE dimension reduction analysis displayed 32 seurat clusters (cluster 0–31) and six bulk labels (B cells, endothelial cells, epithelial cells, fibroblasts, myeloid cells, and NK/T cells). (**C**) Bar plots demonstrated average number (left) and cell proportion (right) of the six types of cells in these samples. (**D**) Cleveland dot plot visualized average expression levels of four classic marker genes CD24, CD44, PROM1, and MKI67 in the six types of cells. (**E**) Expression feature plots revealed the marker genes for cell annotation of the six types of cells. (**F**) Point plot showed marker genes (adjusted *P* < 0.05) of the 32 clusters as Up_Highly (adjusted *P* < 0.01), Down_Highly (adjusted *P* < 0.01), Up_Lowly (adjusted *P* < 0.05), and Down_Lowly (adjusted *P* < 0.05) in a color-coded way. (**G**) Differential expression heatmap illustrated the top five DEGs of the six types of cells. (**H**) Expression feature plots revealing expression of ASPH, CBX2, NOTCH3, HDAC6, HDAC11, MAML1, and NCSTN in the six types of cells. *Abbreviations:* scRNA-seq, single-cell RNA-sequencing; tSNE, t-distributed stochastic neighbor embedding; DEG, differentially expressed genes.

We intended to investigate the clinical profiles and the gene expressions of ASPH, CBX2, NOTCH3, HDAC6, HDAC11, MAML1, and NCSTN in KIRP, so an external validation employing multiple databases was performed. First of all, we found that ASPH, CBX2, NOTCH3, HDAC6, HDAC11, MAML1, and NCSTN were all significantly associated with overall survival in K-M survival analysis (Figure S1). In the database of GEPIA, ASPH, CBX2, NOTCH3, HDAC6, HDAC11, MAML1, and NCSTN were all significantly related to tumor stage (Figure S2). In the LinkedOmics database, CBX2, NOTCH3, HDAC6 and HDAC11 were linked with tumor purity and TNM stage. Besides, ASPH, MAML1, and NCSTN were linked with TNM stage and overall survival (Figure S3, S4). Meanwhile, in the UALCAN database, we also found that ASPH, NOTCH3, HDAC6, MAML1, and NCSTN showed differential expression levels between normal kidney tissues and KIRP tissues. Moreover, expression of ASPH, CBX2, and HDAC6 were associated with histologic subtypes (Figure S5). Employing the Human Protein Atlas database, the protein levels of ASPH, NOTCH3, MAML1, and NCSTN in KIRP tissue were suggested to be higher than those in normal renal tissues, while HDAC6 in KIRP was significantly lower (Figure S6). Last but not least, utilizing scRNA-seq analysis, we revealed that ASPH, CBX2, NOTCH3, HDAC6, HDAC11, MAML1, and NCSTN were co-expressed in fibroblasts and endothelial cells at relatively high levels (Fig. 9).

## Discussion

As one of the malignant genitourinary tumors, KIRP is regarded as a heterogeneous disease in terms of disease progression and patients’ survival [27]. Because of its low occurrence, KIRP is constantly understudied compared to kidney renal clear cell carcinoma (KIRC), making in-depth research for KIRP prognostic prediction challenging [27]. It’s reported that advanced-stage KIRP has no effective treatments currently, and KIRP treatment resistance also appears because of the CSCs [28]. Therefore, the therapy of KIRP is still a problem for oncologists, and there is an urgent need to explore the CSC features of KIRP and find prognostic markers and therapeutic targets for KIRP patients.

In our study, we identified six prognostic SRGs and developed a prediction model that had high reliability (AUC: 0.861). The risk score was shown to be an independent prognostic predictor for patients with KIRP. Additionally, we used Pearson correlation analysis to evaluate SRGs along with corresponding TFs and signaling pathways, in which we discovered that the CBX2 and Notch signaling pathways were most correlated with ASPH. In the meantime, resveratrol was proven to be the most significant potential inhibitor targeting KIRP, which would be of tremendous use in the treatment of KIRP patients.

Chromobox homolog 2 (CBX2), a polycomb repressor complex subunit, was known to be a significant component of Polycomb group (PcG)-mediated repression [29]. In particular, CBX2 assisted polycomb-repressive complex 1 (PRC1) in targeting chromatin by recognizing the repressive mark H3K27me3 [30]. Previous studies revealed that elevated expression of CBX2 was associated with unfavorable survival through retaining CSCs in an undifferentiated state and inhibiting tumor suppressors [30]. What’s more, mounting evidences suggested that CBX2 could block differentiation and promote self-renewal of CSCs, thus playing an important part in tumor initiation and development [31]. For example, CBX2 could drive a cancer stem cell-like phenotype in HCC revealed by multi-omics and multi-cohorts [32]. Similar to our study, there is increasing evidence that overexpression and amplification of CBX2 were substantially related to proliferation, metastasis and poor prognosis in a variety of cancers [33–36]. In hepatoma, an obvious high expression of CBX2 is regarded as an independent poor prognostic factor, and down-regulation of CBX2 expression inhibits the development of liver cancer [37]. Additionally, CBX2 expression in cancer tissues was higher than normal tissues and escalated CBX2 expression was significantly correlated with tumor size, lymph node metastasis, and high TNM stage [29]. Furthermore, Jangal, M. et al. found that inhibiting CBX2 was a promising method to target polycomb complexes in the CSC niche [30].

Aspartate β-hydroxylase (ASPH), a type II transmembrane protein that is approximately 86 kDa, was identified as a part of the family of α- ketoglutarate-dependent dioxygenases [38]. The human ASPH gene lies in q12.3 of chromosome 8 and consists of 214,085 base pairs and 33 exons, which is highly conserved in the evolution of mammals [38]. The entire ASPH is composed of five domains, including a C-terminal catalytic domain, a calcium binding domain, a positively charged luminal domain, a universal transmembrane domain, and an N-terminal cytoplasmic domain [39]. According to the previous study, ASPH catalyzed the hydroxylation of aspartyl and asparaginyl residues of epidermal growth factor (EGF)-like domains that exist in various proteins, Notch receptors and ligands included [40]. Additionally, it was revealed that the overexpression of ASPH had been found in 70–90% of human solid tumors and that ASPH played a significant role in the malignant transformation of solid tumors via promoting proliferation, migration, and invasion of cancer cells [38]. It was worth mentioning that ASPH expression was relatively low or negligible in normal adult tissues but very high in a number of malignancies, including HCC, cholangiocarcinoma, lung, breast, and colon cancer, plus the neoplasms of the nervous system [38, 40]. Besides, ASPH was also overexpressed in pancreatic cancer and played an active part in the regulation of pancreatic cancer cells’ proliferation, migration, and invasion by a variety of signaling pathways [40]. What’s more, in HCC, over-expression of ASPH also resulted in its invasiveness and augmented hydroxylase activity of tumor tissues was associated with unfavorable prognoses of patients with HCC [41]. Therefore, we could draw the conclusion that ASPH might be regarded as a potential prognostic marker in cancer detection [42]. Moreover, ASPH was found to be a potential target for cancer therapy [43] and immunotherapy [44].

In order to further research the deep mechanism of ASPH regulating the development of cancer, we found that the Notch signaling pathway was the overlapped co-expression signal pathway. The Notch signaling pathway was a primordial and remarkably conserved pathway which was related to the communication between contiguous cells through transmembrane ligands and Notch proteins, the single-pass cell surface receptors [38, 45]. As was known to us, the Notch signaling pathway had diverse functions, such as governing embryonic development, the regulation of cell proliferation, differentiation, survival, and apoptosis [40, 46]. What’s more, the Notch signaling pathway also played an active part in the maintenance and self-renewal of stem cells [38].

In recent years, there was increasing evidence that aberrant activation of Notch was involved in the tumor process and inducement of cell proliferation, metastasis, and epithelial-mesenchymal transition in many different solid tumors, which also had translational medical significance [47–50]. Meanwhile, persistent activation of the Notch signaling pathway had been shown to correlate with multiple aspects of cancer biology, such as CSCs, angiogenesis, tumor immunity, and cancer metastasis [45, 46]. When it came to the mechanism between ASPH and the Notch signaling pathway, previous studies revealed that upregulation of ASPH led to enzymatic modification of cbEGF-like repeats in extracellular domains and ligands of the Notch receptor, which enhanced the interaction between the receptor and ligands, as well as the activation of the Notch signaling pathway [38]. For instance, ASPH promoted the interaction between Notch and JAG to keep Notch receptors, ligands, and regulators stable and strengthened ligand-receptor binding, which confirmed that ASPH-Notch axis was critical in carcinogenesis in breast cancer [51]. Furthermore, the ASPH-notch Axis could intricately orchestrate the exosomal transport of prometastatic secretome, thereby facilitating multi-organ metastasis in breast cancer [52]. Additionally, studies had shown that ASPH acted as an intermediate protein that linked upstream growth factor signal cascades with downstream Notch activation, and the activation of the Notch signaling pathway promoted migration, invasion, and metastases in HCC [53]. In general, ASPH could facilitate tumor growth mainly through the activation of the Notch signaling pathway, so it’s possible to block the self-renewal and proliferation of CSC and tumor progression by targeting the Notch signaling pathway [45].

In our study, resveratrol was identified as a potential target drug. Resveratrol, a phytoalexin antioxidant detected in red grapes, was confirmed to play an important part in suppressing various human malignancies, including breast, cervical, uterine, blood, kidney, liver, eye, bladder, thyroid, esophageal, prostate, brain, lung, skin, gastric, colon, head and neck, bone, ovarian, and cervical cancers [54]. Furthermore, mounting evidence suggested that resveratrol affected diverse signal-transduction pathways that controlled inflammation, cell growth and division, apoptosis, metastasis, and angiogenesis and played a critical part in affecting various cancer stages, such as initiation, promotion, and progression [55]. In cervical cancers, resveratrol could concurrently inhibit STAT3, Wnt, and Notch signaling activations, leading to the growth arrest and apoptosis of cervical squamous cell carcinoma and adenocarcinoma cells [56]. In human T-cell acute lymphoblastic leukemia, resveratrol triggered apoptosis through suppressing the Notch signaling pathway along with the downstream effectors and regulating the operation of interacting apoptosis pathways, which were mediated by p53 and PI3K/Akt [57]. On the contrary, some research revealed that resveratrol repressed cell growth and enhanced redifferentiation of anaplastic thyroid carcinoma cells through the activation of Notch1 signaling [58]. The mechanisms of Notch signaling regulated by resveratrol appeared to be contradictory, but the comprehensive mechanisms revealed that the effects of resveratrol on the Notch signaling pathway were context-dependent. Notch signaling was effectively inhibited by resveratrol when it was oncogenic, but it was activated when it acted towards tumor-suppression [59]. Generally speaking, resveratrol inhibited cancer cell growth via a variety of important pathways and could be regarded as a promising anti-cancer agent in the future.

Our investigation nevertheless had several inevitable limitations. Firstly, this was merely bioinformatics research, no direct mechanism experiments had previously proven the scientific hypothesis. Secondly, despite the external validation performed by multiple databases, the clinicopathological feature analysis of the data was incomplete and the sample size was limited. Thirdly, potential statistical bias was unavoidable because of the heterogeneity of data from different batches. Finally, the only single omics analysis was an intrinsic restriction to our work. However, based on this scientific hypothesis, hopefully we will perform further cell and animal experiments to verify the direct mechanism. Furthermore, the relationship between CBX2, ASPH, Notch signaling pathway, and KIRP tumorigenesis and development will be clarified in the future. These biological experiment assays might contribute to identifying novel prognostic factors and potential therapeutic targets in KIRP.

## Conclusions

According to our comprehensive bioinformatics results, we hypothesized that transcription factor CBX2 regulated ASPH may well be correlated with the carcinogenesis, cancer development, and unfavorable prognosis of KIRP through activation of Notch signaling pathway.

### Electronic supplementary material

Below is the link to the electronic supplementary material.

Supplementary Material 1

## Data Availability

All datasets for this study are included in the TCGA-KRIP program (https://portal.gdc.cancer.gov).

## Code availability

All data was processed by Perl (Practical Extraction and Report Language, https://www.perl.org), R software version 3.6.1 (www.r-project.org; Institute for Statistics and Mathematics, Vienna, Austria) and packages acquired from Bioconductor (http://www.bioconductor.org/packages/release/bioc/html/impute.html).
