# Supplementary material for: Identification of prognostic stemness-related genes in kidney renal papillary cell carcinoma
Source: BMC Med Genomics. 2024 May 3;17:121. doi: 10.1186/s12920-024-01870-2 (PMC11067181; doi:10.1186/s12920-024-01870-2)
Supplement: Supplementary file 1 — Supplementary Material 1 [file 12920_2024_1870_MOESM1_ESM.docx]

**Supplementary materials**


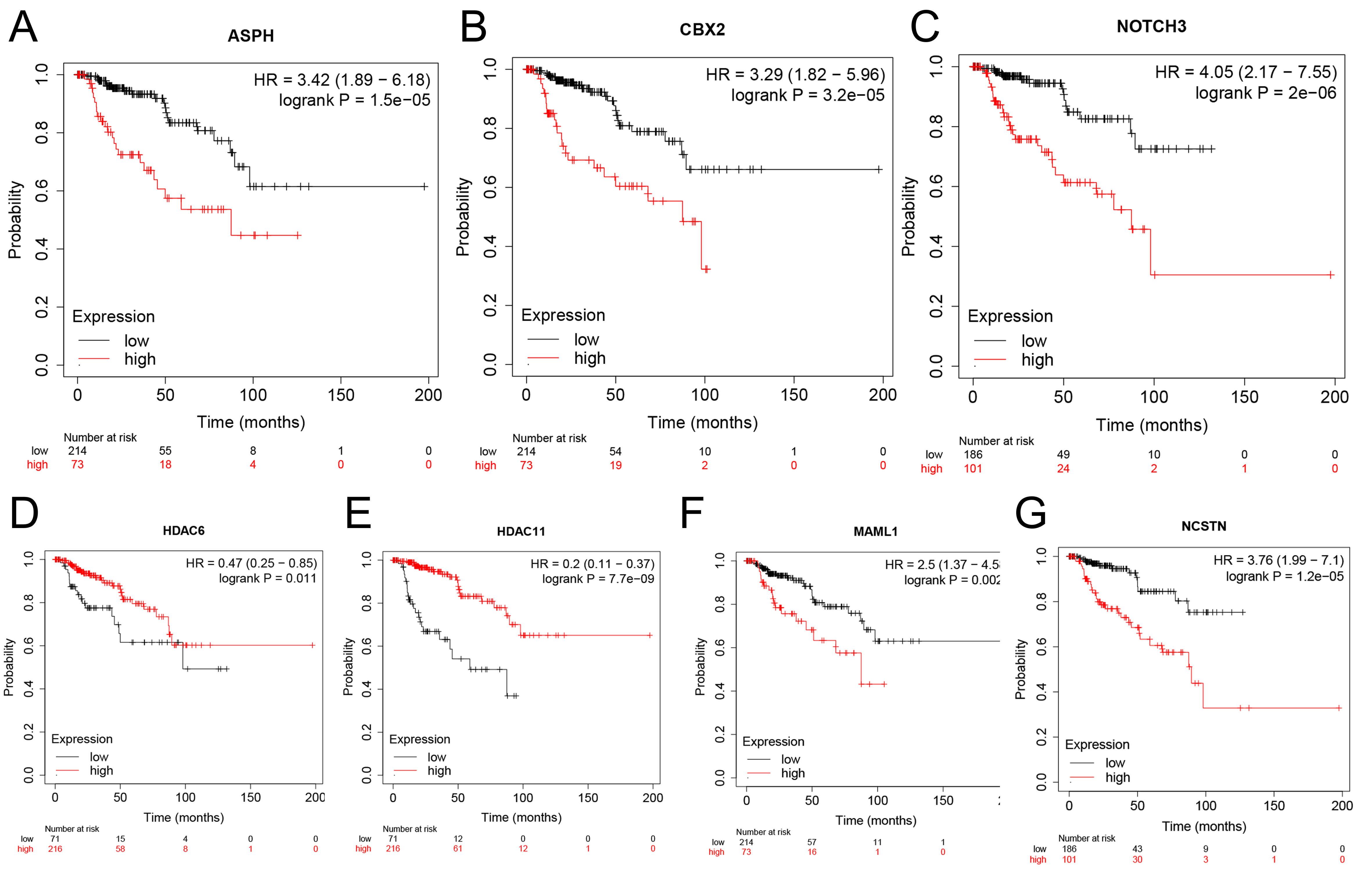


**Figure S1.** The Kaplan-Meier curve of ASPH, CBX2, NOTCH3, HDAC6, HDAC11, MAML1 and NCSTN (A-G) in KIRP patients using K-M Plotter database.


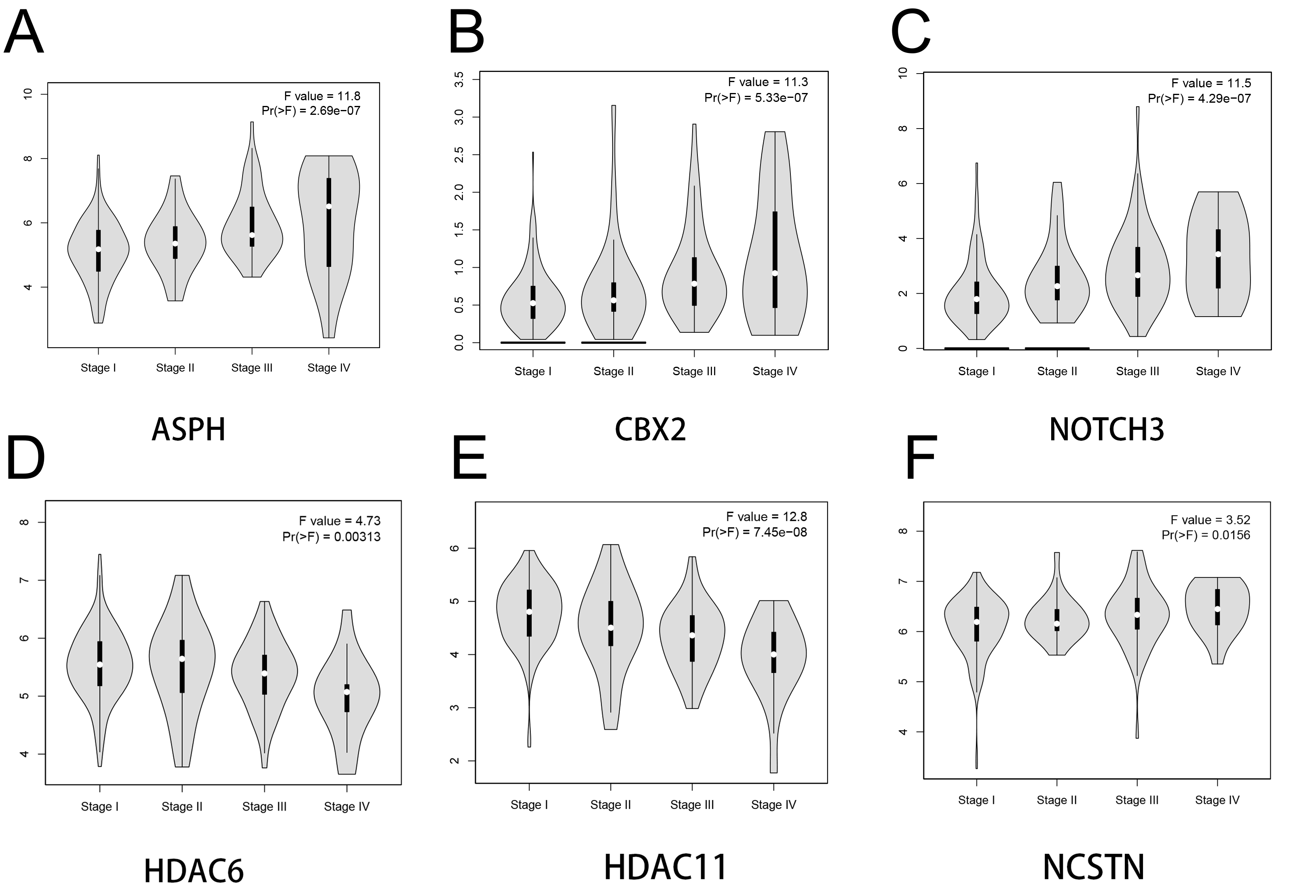


**Figure S2.** The stage profiles of ASPH, CBX2, NOTCH3, HDAC6, HDAC11 and NCSTN (A-F) in KIRP patients in GEPIA database.


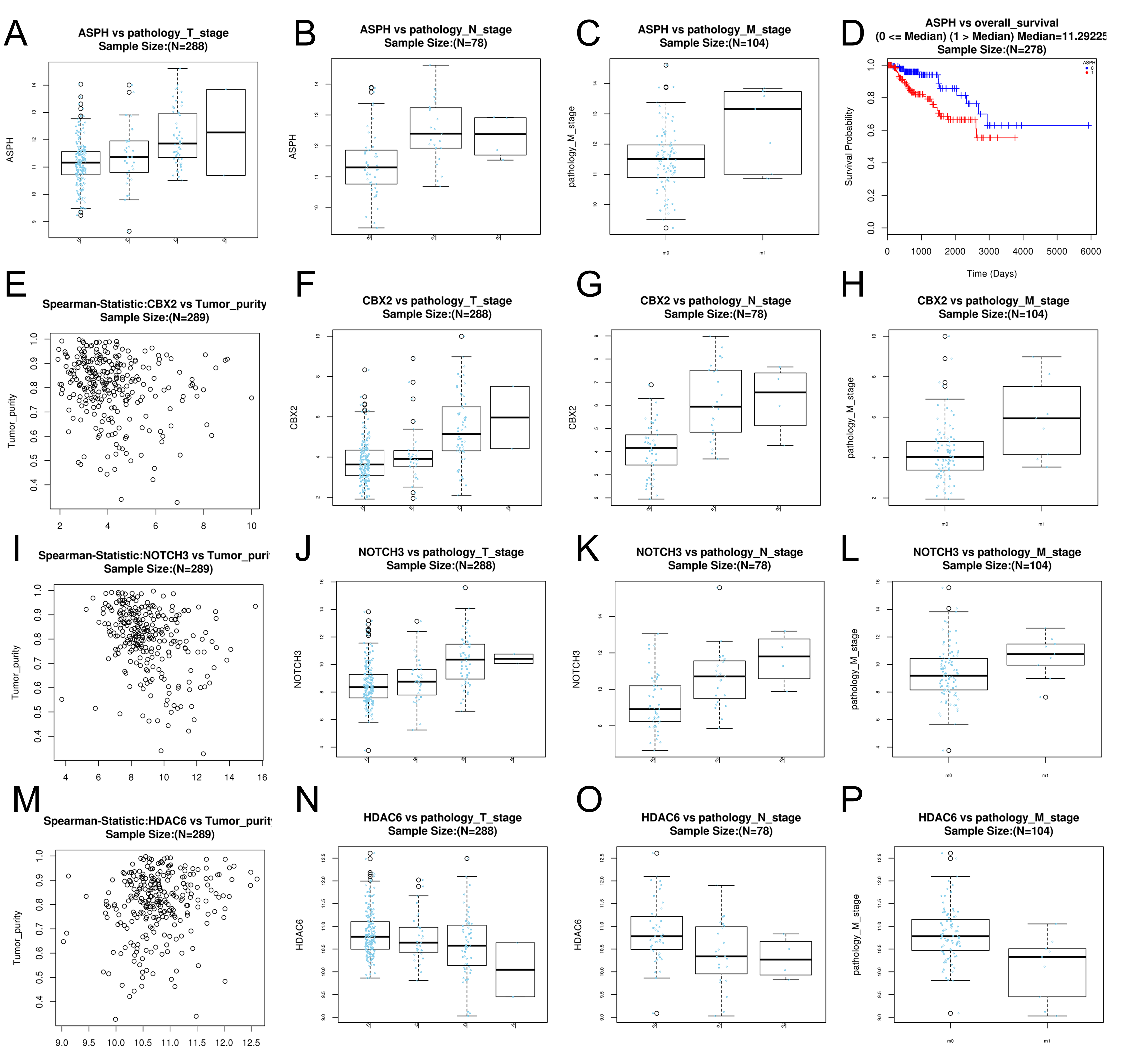


**Figure S3.** Integrative analysis of clinical profiles using LinkedOmics database, ASPH (A-D), CBX2 (E-H), NOTCH3 (I-L), HDAC6 (M-P).


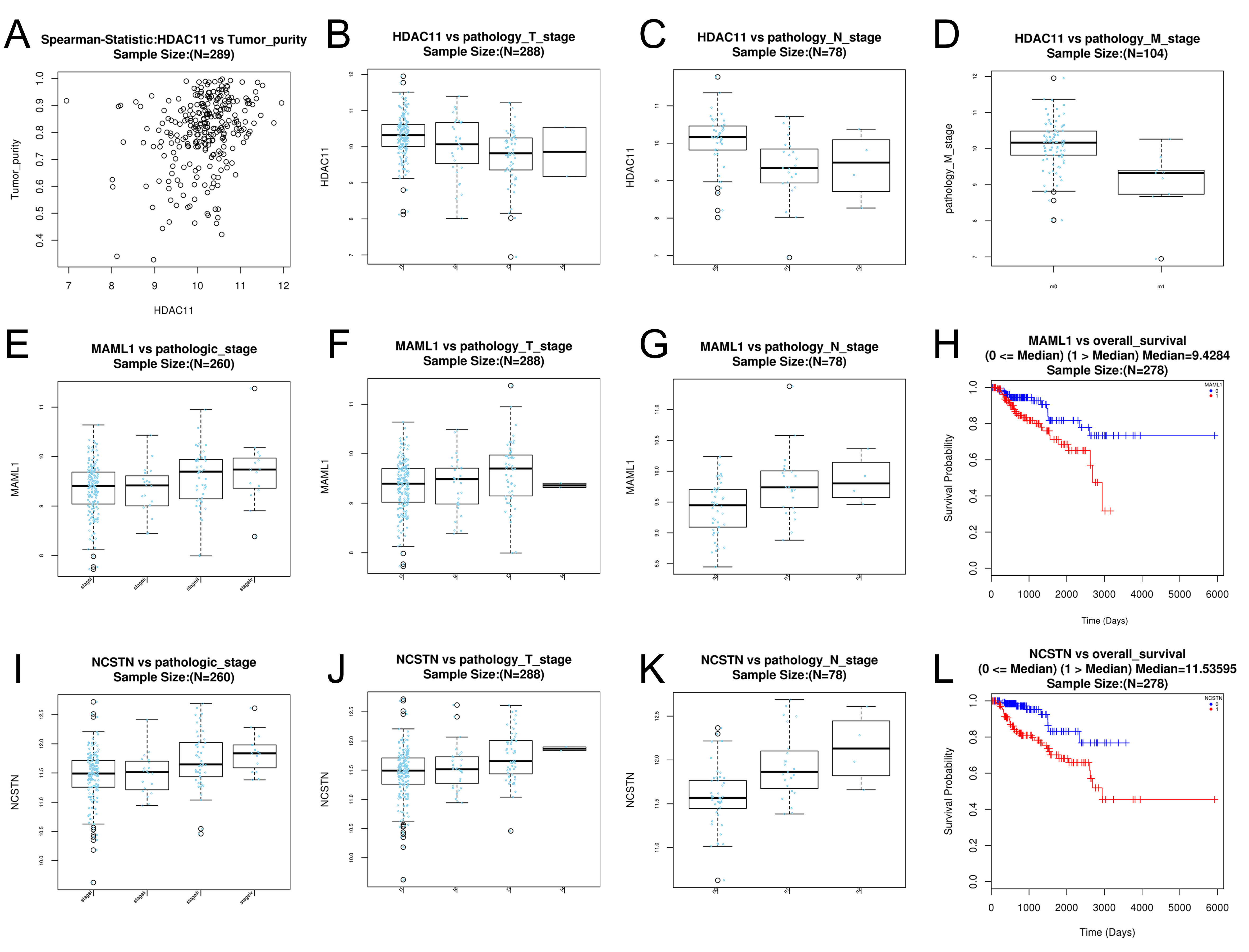


**Figure S4.** Integrative analysis of clinical profiles using LinkedOmics database, HDAC11 (A-D), MAML1 (E-H), NCSTN (I-L).


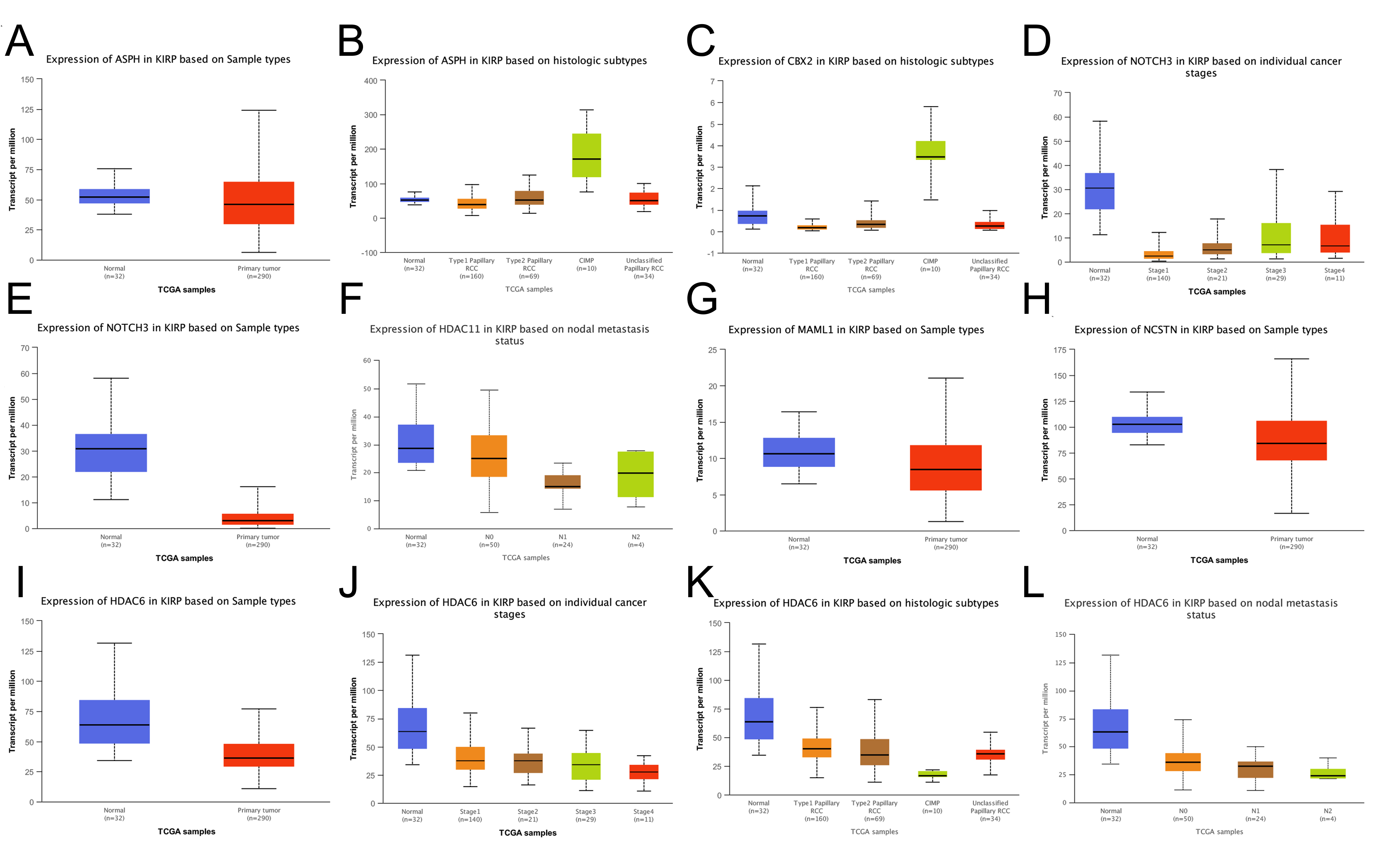


**Figure S5.** The expression levels of ASPF (A, B), CBX2 (C), NOTCH3 (D, E), HDAC6 (I-L) HDAC11 (F), MAML1 (G)and NCSTN (H) in KIRP.


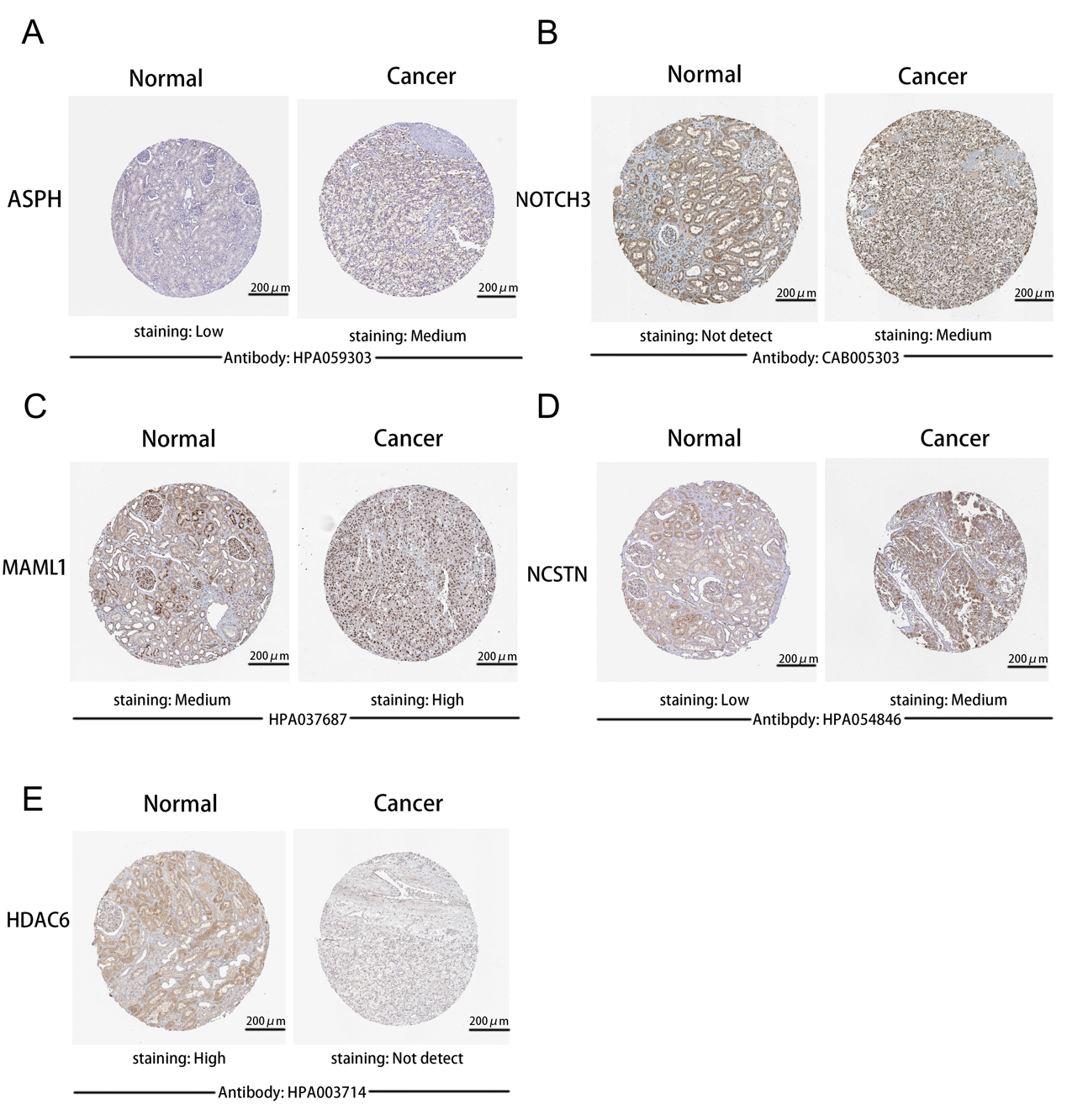


**Figure S6.** Validation of ASPH, NOTCH3, MAML1, NCSTN, HDAC6 on a translational level between normal kidney and KIRP in the Human Protein Atlas database.
